# Light-Induced Changes within Photosystem II Protects *Microcoleus* sp. in Biological Desert Sand Crusts against Excess Light

**DOI:** 10.1371/journal.pone.0011000

**Published:** 2010-06-08

**Authors:** Itzhak Ohad, Hagai Raanan, Nir Keren, Dan Tchernov, Aaron Kaplan

**Affiliations:** 1 Department of Biological Chemistry, The Hebrew University of Jerusalem, Jerusalem, Irael; 2 Department of Plant and Environmental Sciences, The Hebrew University of Jerusalem, Jerusalem, Israel; 3 The Interuniversity Institute for Marine Sciences in Eilat, The Hebrew University of Jerusalem, Jerusalem, Israel; University of Wisconsin-Milwaukee, United States of America

## Abstract

The filamentous cyanobacterium *Microcoleus vaginatus*, a major primary producer in desert biological sand crusts, is exposed to frequent hydration (by early morning dew) followed by desiccation during potentially damaging excess light conditions. Nevertheless, its photosynthetic machinery is hardly affected by high light, unlike “model” organisms whereby light-induced oxidative stress leads to photoinactivation of the oxygen-evolving photosystem II (PSII). Field experiments showed a dramatic decline in the fluorescence yield with rising light intensity in both drying and artificially maintained wet plots. Laboratory experiments showed that, contrary to “model” organisms, photosynthesis persists in *Microcoleus* sp. even at light intensities 2–3 times higher than required to saturate oxygen evolution. This is despite an extensive loss (85–90%) of variable fluorescence and thermoluminescence, representing radiative PSII charge recombination that promotes the generation of damaging singlet oxygen. Light induced loss of variable fluorescence is not inhibited by the electron transfer inhibitors 3-(3,4-dichlorophenyl)-1,1-dimethylurea (DCMU), 2,5-dibromo-3-methyl-6-isopropylbenzoquinone (DBMIB), nor the uncoupler carbonyl cyanide-p-trifluoromethoxyphenylhydrazone (FCCP), thus indicating that reduction of plastoquinone or O_2_, or lumen acidification essential for non-photochemical quenching (NPQ) are not involved. The rate of Q_A_^−^ re-oxidation in the presence of DCMU is enhanced with time and intensity of illumination. The difference in temperatures required for maximal thermoluminescence emissions from S_2_/Q_A_^−^ (Q band, 22°C) and S_2,3_/Q_B_^−^ (B band, 25°C) charge recombinations is considerably smaller in *Microcoleus* as compared to “model” photosynthetic organisms, thus indicating a significant alteration of the S_2_/Q_A_^−^ redox potential. We propose that enhancement of non-radiative charge recombination with rising light intensity may reduce harmful radiative recombination events thereby lowering ^1^O_2_ generation and oxidative photodamage under excess illumination. This effective photo-protective mechanism was apparently lost during the evolution from the ancestor cyanobacteria to the higher plant chloroplast.

## Introduction

The desiccation-tolerant filamentous cyanobacterium *Microcoleus vaginatus* (hereafter *Microcoleus*) is the main primary producer in desert biological sand crusts, where it is exposed to strong illumination, limiting moisture and nutrients and large variations in temperature [1]. The field experiments presented here and isolation of the axenic *Microcoleus* strain used in our laboratory studies were performed in the Nizzana region, NW Negev, Israel where dew is a major source of moisture to the organisms inhabiting the biological crusts. Information on the climatic conditions in this region is available [2]. Light-driven oxidative damage to core components of photosystem II (PSII) in oxygenic photo-autotrophs is the primary cause of the decline in its activity (see [3],[4],[5] and references therein). Back electron flow to pheophytin (hereafter, Pheo) and radiative charge recombination of P_680_ **^+^/**Pheo^−^ in PSII generates ^1^O_2_ [6] due to the interaction of ground-state triplet dioxygen with excited triplet chlorophyll, which leads to photoinactivation of PSII (as well as oxidative stress extending beyond the thylakoids or chloroplast limits). The rates of back electron flow to the oxidized primary electron donor, P^+^, and charge recombination *via* Pheo, increase if the rate of electron flow exceeds that of photosystem I and carbon fixation [3],[5],[7].

Dissipation of excess light excitation as heat *via* modulation of energy transfer from antennae to the reaction centers (non-photochemical quenching, NPQ) provides partial protection against light-induced damage to PSII [8],[9]. Processes leading to reaction center quenching were also implicated in the protection against photoinhibition ([5] and references therein). In cyanobacteria, the qT component of NPQ operates to some extent in the phycobilisomes light-harvesting antenna [10]. The modulation of this process might possibly involve reversible phycobilisomes attachment to PSII [11],[12]. In addition, NPQ, ascribed to the carotene-binding OCP protein, is involved in energy dissipation at the phycobilisomes core [13].

Earlier studies showed that rewetting of a recently dehydrated crust resulted in a complete recovery of the photosynthetic activity, measured by fluorescence, despite the very high light intensity experienced by the cells during desiccation and subsequently [14],[15]. These observations prompted us to study the mechanisms whereby *Microcoleus* is able to withstand these conditions without severe damage to its photosynthetic machinery. Recently, a model was presented whereby non-radiative charge recombination could help to reduce potential photodamage to reaction center II [5]. Our study demonstrates a reversible, drastic decrease of PSII radiative charge recombination that is triggered by exposure of *Microcoleus* to light intensities exceeding saturation of oxygen evolution, thus conferring protection against light-induced oxidative stress.

## Results

### Fluorescence yield in natural biological sand crusts

**Figure 1:**
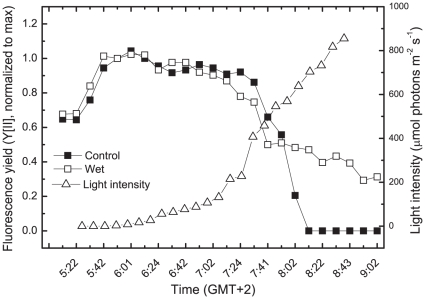
Fluorescence yield in *Microcoleus* inhabiting desert sand crusts as affected by time and moisture. Fluorescence emitted from 4 crust samples was measured using a PAM 2500 (Walz, Effertlich, Germany). Two plates were sprayed with water to prevent dehydration. The experiments were repeated 4 times with minor variations in the results.

Chlorophyll fluorescence yield is often used to assess photosynthetic performance and even to calculate a relative electron transfer rate. Fluorescence has also been used to assess the photosynthetic activity in lichens [16] and desert soil crusts [17]. To assess the activity of *Microcoleus* in the dry desert crust of Nizzana, where it is the main primary producer, we measured the fluorescence yield as affected by the moisture and time from dawn (**Fig. 1**). In 12 independent experiments, the fluorescence began to rise already in the dark after rewetting [14] and declined when the light intensity was about 200 µmol photons m^−2^s^−1^ at about 0720. This decline (at least 50%) was observed regardless of whether the crust samples were maintained hydrated by water spraying or allowed to dry out after the early morning dew (the sole water source during most of the year). An additional decline in fluorescence yield was observed in drying crusts that were not provided with additional water, presumably due the arrest of photochemical activity. The decline in fluorescence in the drying crust does not reflect sustained photoinhibitory damage to PSII since full fluorescence emission could be recovered shortly after rewetting [14]. Due to the relatively low abundance of photosynthetic organisms in natural crusts, particularly in arid regions, we were unable to measure changes in O_2_ or CO_2_ exchange and hence could not determine whether the decline in fluorescence when the light intensity reached about 200 µmol photons m^−2^s^−1^ reflects a decreasing rate of photosynthesis in parallel with loss of fluorescence yield.

### Fluorescence decline in crusts does not involve concomitant reduction of O_2_ evolution

**Figure 2:**
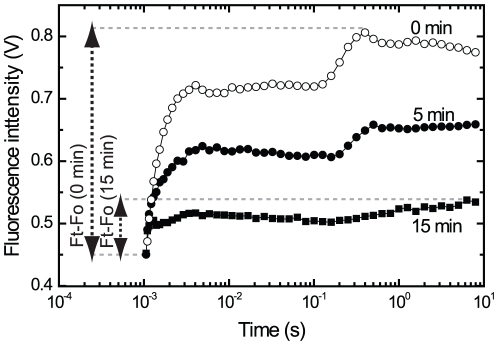
The fluorescence kinetics from dark-adapted and excess light treated *Microcoleus* cells as measured by FL3000 fluorimeter. Cells (corresponding to 10 µg chl/ml) were exposed to 1800 µmol photons m^−2^ s^−1^ of white light (optical path 2.2 cm) for various durations after 2 min dark adaptation period. The fluorescence intensity (0.5 cm optical path) is expressed in volts. The first data point, one msec after the onset of actinic blue and orange light, is defined as the Fo. The maximal fluorescence value in each trace is defined as Ft. In cases where DCMU was used, Ft = Fm and Ft–Fm = Fv.

To overcome our inability to measure CO_2_ or O_2_ exchange in the natural crusts, we used artificial crusts where *Microcoleus* suspensions were soaked onto nitrocellulose filters (see [15]) that were maintained wet during exposure to 500 µmol photons m^−2^s^−1^ of blue and orange light. The light intensity used here is higher than experienced in the natural crusts at the point (200 µmol photons m^−2^s^−1^) where the fluorescence started to decline, since the amount of *Microcoleus* filaments on the artificial crusts is much higher (to enable measurements of O_2_ evolution). This treatment resulted in a gradual loss of fluorescence to 18–23% (n = 4) of its initial value within 25 min. The artificial crusts were then quickly dispersed and transferred to the O_2_ electrode. The measurements showed that the rate of O_2_ evolution declined only to 85–92% (n = 4) of its initial value during the 25 min exposure, i.e. much less than variable fluorescence. These data indicated uncoupling between variable fluorescence values and the photosynthetic electron transport. In Figure **2** we provide an example of the raw fluorescence data measured before and after exposure of *Microcoleus* to excess light. The fluorescence parameters presented in the following figures were calculated as shown here, unless otherwise noted.

### Resistance of photosynthetic oxygen evolution to excess light

**Figure 3:**
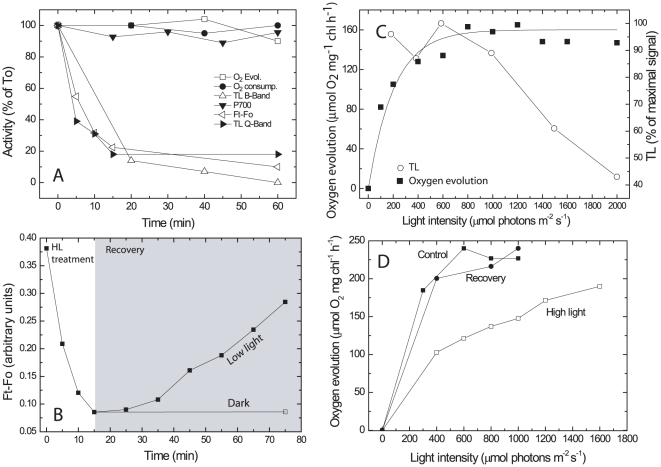
Effect of excess light treatment on electron flow parameters. **A**. *Microcoleus* cells (10 µg chl ml^−1^, optical path 1.5 cm) were exposed to 2000 µmol photons m^−2^ s^−1^ for different durations and the various parameters were measured at indicated times. In different experiments the 100% values corresponded to 160–270 µmol O_2_ mg chl^−1^ h^−1^ evolved in light and 40–100 µmol O_2_ mg chl^−1^ h^−1^ taken up in darkness; Maximal Ft–Fo at time zero of excess illumination was 0.37, measured using a FL3000 fluorimeter (see Fig. 2). TL, 9000–11,000 photons s^−1^; P700 oxidation −3×10^−4^ Δ*I*/*I* (TL measurements were performed after a saturating flash provided at −22°C; the maximal B-band emission was at 25°C). **B.** Cell suspension (10 µg chlorophyll ml^−1^) was exposed to 1200 µmol photons m^−2^ s^−1^ for 17 min to trigger loss of fluorescence (Ft–Fo) as measured by the FL3000 fluorimeter. The light intensity was then reduced to 50 µmol photons m^−2^ s^−1^ or darkness and recovery of fluorescence emission was measured for 60 min. **C.** Cells (12 µg chl ml^−1^) were exposed to various light intensities for 17 min followed by measurements of O_2_ evolution and TL B band emission. **D.** The rate of O_2_ evolution as a function of light intensity by control and cells exposed for 17 min to 2000 µmol photons m^−2^ s^−1^ and followed by 50 min recovery at 70 µmol photons m^−2^ s^−1^. **Note**: Each experiment presented here was performed several times using independent cell cultures. The patterns of the results presented are characteristic examples, however standard deviation values were not plotted.

Uncoupling between fluorescence parameters and O_2_ evolution may invalidate the use of fluorescence to assess the rates of electron transport, at least in *Microcoleus*. To study the mechanism involved we examined the relationship between photosynthetic activities and fluorescence yield in laboratory-grown liquid cultures of *Microcoleus*. In the crusts the *Microcoleus* filaments are exposed to very high light intensity, 10-fold higher than those which led to the initiation of fluorescence decline. To study the response of photosynthetic activity to excess light intensities, we measured simultaneously CO_2_-dependent O_2_ evolution, chlorophyll fluorescence (Ft–Fo) and thermoluminescence emission (TL). The TL emission results from charge recombination of S_2,3_/Q_B_^−^ or S_2_/Q_A_^−^ in the presence of DCMU, designated B and Q bands, respectively (see scheme in Materials and Methods). We also determined the rate of dark respiration (O_2_ uptake) and photosystem I (PSI) oxido-reduction activity. *Microcoleus* cells were exposed to 2000 µmol photons m^−2^ s^−1^ (about 3 times higher than required to saturate the rate of oxygen evolution with the light path across the O_2_ electrode and the cell densities used) for 1 h. As indicated, this intensity is considerably higher than measured when the fluorescence started to decline in the field experiments (**Fig. 1**). However, the cell densities used in the laboratory experiments and optical path are considerably higher than in the crusts. Furthermore, the light path in the O_2_ electrode (2.2 cm) is much longer than the width of the *Microcoleus* layer in the natural crusts (it varies from less than 1 mm in relatively dry areas, used here (**Fig. 1**), to a few mm in more humid places where many other organisms are also present). Samples were taken at time intervals and dark-adapted for 2 min before the measurements (**Fig. 3A**). Oxygen evolution, respiration and PSI activity were not affected by the excess light treatment whereas chlorophyll fluorescence and TL bands emission declined within 25 min by up to 85% and 90%, respectively. The fact that O_2_ evolution was not affected indicated that loss of fluorescence and TL emission does not reflect inhibition of forward electron flow activity of *Microcoleus* PSII. The loss of Ft–Fo, which resembled that in the natural and artificial crusts, did not involve a significant change in the Fo level. Interestingly, the fluorescence signal recovered slowly, about 75% within 70 min when the cells were maintained at low light intensity (50 µmol photons m^−2^s^−1^), but *not* in darkness (**Fig. 3B**). The mechanism involved in the slow recovery is being investigated; its kinetics do not support antenna events, which are usually faster [8].

### Light intensity dependence of fluorescence and TL decline

To the best of our knowledge this is the first report of extensive loss of Fv and TL signals induced by exposure to excess light while the rate of oxygen evolution was hardly affected. Optimally, a mechanism for energy dissipation should be activated only at light intensities close to those required to saturate forward electron flow and CO_2_ fixation. Accordingly we measured the TL B band signal intensity and maximal O_2_ evolution as affected by 17 min of illumination at different light intensities. This duration was selected since most of the TL decline was already detected by this time (**Fig. 3A**). Oxygen evolution reached a maximum at about 700 µmol photons m^−2^ s^−1^ and remained essentially constant with rising light intensities whereas the TL signal severely declined (**Fig. 3C**).

A decline in chlorophyll fluorescence and TL where O_2_ evolution is not affected (**Fig. 3**A) could result from different processes, or a combination of processes, including a large surplus of PSII activity over carboxylation capacity, non-photochemical quenching (NPQ) in the antenna, loss of radiative charge recombination and/or induction of non-radiative P**^+^**/Q_A_ **^−^** charge recombination [5],[18],[19],[20] and reaction center quenching [21]. The mechanism involved is triggered by light intensities close to those saturating oxygen evolution (**Fig. 3C**).

We applied the electron acceptors from PSII 2,6-dimethylbenzoquinone (DMBQ) and,2,6-dichlorophenolindophenol (DCPIP), to measure PSII activity directly on various independent cultures. The maximal discrepancy we could recover was 10–30% higher PSII activity than the maximal rate of CO_2_-dependent O_2_ evolution measured on the same cultures, i.e. too small to account for the stability of O_2_ evolution, certainly during extended photoinhibitory conditions.

**Figure 4:**
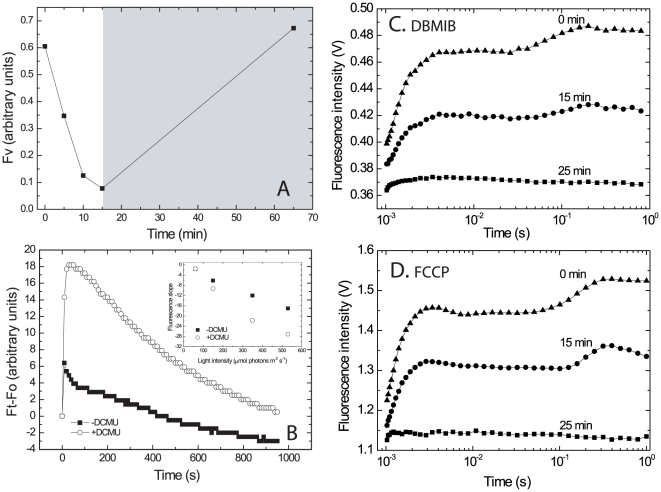
Loss of variable fluorescence, even in the presence of DCMU, DBMIB and FCCP following excess light treatment. **A.** Cells suspensions (7.5 µg chl ml^−1^) were exposed to 2000 µmol photons m^−2^ s^−1^, 2.2 cm optical path, for the indicated times in the presence of 10 µM DCMU, a concentration which completely blocked CO_2_-dependent O_2_ evolution. Fv was measured after dark adaptation for 2 min using the FL 3000 fluorimeter; optical path 0.5 cm. After 15 min of excess illumination the light intensity was reduced to 50 µmol photons m^−2^ s^−1^ for 50 min. **B.** Fluorescence emission kinetics of dark-adapted cells in the presence or absence of 10 µM DCMU. The cells were exposed for 950 s to 530 µmol photons m^−2^ s^−1^ of blue light using the IMAG-MAX PAM. Note that in its setup, this light intensity is the maximal but it is not saturating. **Insert:** Rate of fluorescence decline at the 150–900 s range for cultures exposed to varying light intensities (100–530 µmol photons m^−2^ s^−1^). **C**. The experiment was performed as in (A), 2000 µmol photons m^−2^ s^−1^, 2.2 cm optical path, but with 10 µg chl ml^−1^ and 0.15 µM DBMIB instead of DCMU. Note that DBMIB itself is a fluorescence quencher and the procedure used to minimize this effect is explained in the Materials and Methods section. **D.** Experiment performed as in (C) but in the presence of 10 µM FCCP.

A decline in the energy transfer from the phycobilisomes to PSII in response to excess light could lead to lower chlorophyll fluorescence and a reduced rate of O_2_ evolution at limiting light without affecting the maximal O_2_ evolution at saturating light intensity [11]. Changes in the energy transfer from the phycobilisomes to PSII were assessed from the ratio of PSII to phycobilisomes emissions at 77K (683 nm and 650 nm, respectively) following excitation at 620 nm (maximal absorbance of the phycobilisomes). A 2000 µmol photons m^−2^ s^−1^ for 17 min pretreatment, as in Fig. 3A, resulted in 80% loss of Ft-Fo but only a 16% decline in PSII/phycobilisomes originated emissions (not shown). A massive rise in the fluorescence emitted by the phycobilisomes, as in the case of *Synechocystis* WH7803 [11], was not observed. These data and those presented in Fig. 4, below, did not lend support to the possibility that the decline in fluorescence is driven by energy quenching in the antenna of PSII. On the other hand, the same light treatment led to a marked decline in the rate of O_2_ evolution at limiting but not at saturating light intensity (**Fig. 3D**). Since, as indicated, the decline in the energy transfer efficiency from the phycobilisomes is relatively small, the decreased O_2_ evolution at limiting light is attributed to a change in the *Microcoleus* reaction center due to the excess light treatment. When the light intensity was lowered, allowing recovery of Fv (**Fig. 3B**), the rate of oxygen evolution at limiting light intensities increased to values similar to those observed in control, untreated cells (**Fig. 3D**).

### The process leading to fluorescence decline does not require reduction of the plastoquinone pool

The redox state of the plastoquinone pool may modulate reduction of oxygen [22],[23]. Furthermore, a correlation between inactivation of plastoquinone reduction (due to photoinactivation of PSII) and phycobilisomes coupling has been demonstrated in *Synechocystis* [24]. We examined the effect of 10 µM DCMU, which completely abolished O_2_ evolution and thus the reduction of the plastoquinone pool by PSII on the light-induced loss of Fv (Fm–Fo, as defined in the legend of Fig. 2). The cells were dark-adapted for 2 min in the presence of DCMU, exposed to 2000 µmol photons m^−2^ s^−1^ for various durations (0–15 min), followed by measurements of the fluorescence intensity (**Fig. 4**A). The fluorescence declined significantly in response to the excess light in both the absence (**Fig. 3A and B**) and the presence of DCMU (**Fig. 4A**). A complete recovery of the fluorescence was observed when the cells were maintained under 50 µmol photons m^−2^ s^−1^ for 50 min as in Fig. 3B. Naturally, the emitted fluorescence was significantly higher in DCMU-treated cells (**Fig. 4B**) and it reached a maximum after 20–50 s regardless of DCMU presence, but dropped significantly thereafter although the excess light treatment continued (**Fig. 4B**). In Fig. 4B we present data obtained using continuous, sub-saturating blue light excitation where the contribution of the phycobilisomes antennae to fluorescence emission is negligible; similar data were obtained using both orange and blue light (**Fig. 4A**).

To examine whether a cyclic PSI electron transport is involved in the observed fluorescence decline, even in the presence of DCMU, we applied 2,5-dibromo-3-methyl-6-isopropylbenzoquinone (DBMIB) which inhibits electron transport at the cytochrome complex, and thus both the linear and PSI cyclic electron routes. The data (**Fig**. **4C**) clearly show that exposure to excess light in the presence of DBMIB resulted in a large drop in the fluorescence emission similarly to the decline in control cells exposed to excess light (Figs. 2 and 3A). It should be noted that DBMIB itself is known to quench fluorescence. Thus, to avoid possible artifacts, we examined a range of DBMIB concentrations and used 0.15 µM, which showed minimal fluorescence quenching in dark-adapted cells but nevertheless completely inhibited O_2_ evolution (see Materials and Method). Application of the uncoupler carbonyl cyanide-p-trifluoromethoxyphenylhydrazone (FCCP) resulted in severe inhibition of O_2_ evolution but no effect on respiration. Nevertheless, a large decline in fluorescence was observed in excess light treated cells (Fig. 4D). The fluorescence decline was similar in size to that observed in DCMU or DBMIB treated cells suggesting that thylakoid lumen acidification, such as essential to activate NPQ, is not involved in the excess light driven fluorescence decline. These results demonstrate that continuous exposure to excess light induces a significant but reversible loss of fluorescence emission, even when electron transport past Q_A_ and lumen acidification are inhibited by the presence of DCMU, DBMIB or FCCP.

### Excess light does not stimulate oxygen consumption

Reduction of oxygen *via* a PSII cyclic electron flow that requires participation of PQH_2_ oxidases (PTOX) was reported in several cases [22],[23]. Since DCMU and DBMIB, which block the reduction of plastoquinone, did not inhibit the excess light-induced fluorescence loss in *Microcoleus* (**Fig. 4**), it is also unlikely that the fluorescence decline involves accelerated oxygen reduction. Nevertheless, we examined this possibility by applying a membrane inlet mass spectrometer whereby O_2_ uptake (including respiration) can be distinguished from O_2_ evolution using stable oxygen isotopes [25]. The extent of light dependent ^18^O_2_ uptake relative to water splitting in PSII (assessed from ^16^O_2_ production) was measured on cells that were exposed to either 30 or 1500 µmol photons m^−2^ s^−1^ at 25°C. Gross O_2_ evolution was assessed from ^16^O_2_ formation and uptake of ^18^O_2_ was used to measure oxygen consumption. The rate of ^18^O_2_ decline in the presence of DCMU was identical to that observed in darkness and was not affected by the light intensity. The same concentration of DCMU severely inhibited O_2_ evolution but not the decline in fluorescence. These data do not support a significant cyclic electron transfer in PSII of *Microcoleus* with O_2_ serving as the electron acceptor, and reduction of plastoquinone by dioxygen cannot be involved in the loss of fluorescence.

### Thermoluminescence parameters

**Figure 5:**
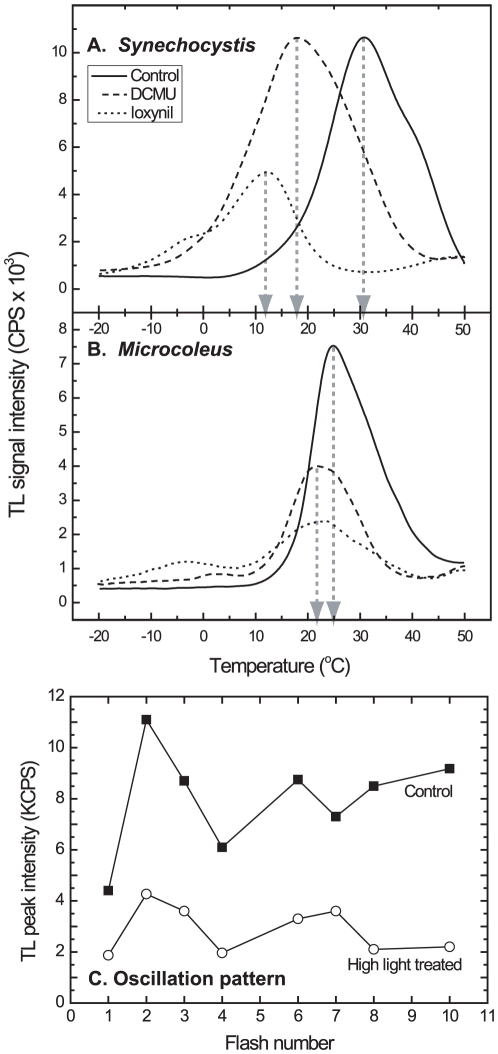
Temperature-dependence of TL emissions of *Synechocysti*s (A) and *Microcoleus* cells (B). Measurements were carried out as described in Materials and Methods. Note the significantly smaller difference in the maximal Q and B bands temperatures in *Microcoleus* as compared to those observed in *Synechocystis* used as a “model organism”. The intensities of the Q bands are partially quenched by the herbicides used [29].

Excess light induced loss of TL emission (**Figs. 3A and C**), uncoupling of variable fluorescence from electron transport (measured as O_2_ evolution), and a decline in Fv even in the presence of DCMU (**Fig. 4**). On the other hand, excess light caused a relatively small rise in fluorescence emitted from the phycobilisomes at 77^o^K (not shown). Taken together the data suggested reversible alterations in the PSII core. We examined whether the excess light treatment induced changes in the energy required for the recombination of S_2,3_/Q_B_^−^→S_1,2_/Q_B_ (B band emission) and S_2_/Q_A_^−^→S_1_/Q_A_ (Q band emission) redox pairs (see [19],[20],[26] and the scheme in Materials and Methods). In “model” organisms such as *Synechocystis* sp. strain PCC 6803 (hereafter *Synechocystis*, **Fig. 5A**), green algae and higher plants [27],[28] the maximal B bands emissions were detected at 30–35°C. The Q band obtained in the presence of various herbicides that block electron flow *via* the Q_B_ site occurs at significantly lower temperatures in higher plants [28]. The Q band of cyanobacteria such as *Synechocystis* occurs at 17°C or 12°C in the presence of DCMU or ioxynil, respectively (**Fig. 5A**). These differences between the B band and Q band temperatures are attributed to changes in the properties of the Q_B_ site due to binding of various types of herbicides that affect Q_A_/Q_B_ interaction [29]. Contrary to the “model organisms”, in 16 independent experiments on *Microcoleus* the average Q band peak was 22±2.6°C and the average B band peak was 26±2.7°C (**Fig. 5B**). Thus, the average difference in peak temperatures (3.5°C±1.8) between the Q and B bands in *Microcoleus* is significantly smaller than observed in “model organisms”, indicating higher activation energy for radiative recombination between Q and Pheo. Furthermore, the maximum temperature of the Q band (22°C) was hardly affected by the type of herbicide used (**Fig. 5B**), indicating a difference in the interaction of Q_A_/Q_B_ sites from that observed in “model organisms”. Exposure to excess light led to a significant decline of the TL signal intensity (**Figs.** **3A, 3C** and **5C**). Figure **5C** also shows that *Microcoleus* exhibited the classical TL oscillation pattern (maximal TL at the 2^nd^ and 6^th^ flashes) but their intensity and maximal emission pattern declined after exposure to excess light. This decline most probably reflects a decreased flow of electrons in the radiative recombination pathway with time of exposure to excess light.

### Kinetics of Q_A_^−^ re-oxidation in cells exposed to high light

**Figure 6:**
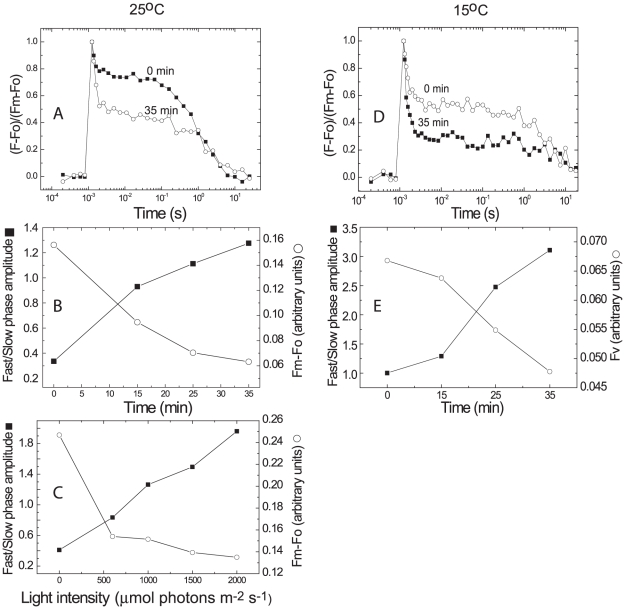
Kinetics of Q_A_^−^ oxidation in the presence of DCMU following exposure to excess light. **A–C.** Cell suspensions (7.5 µg chl ml^−1^) were exposed to 2000 µmol photons m^−2^ s^−1^ for 0–35 min, at 25°C. Q_A_^−^ oxidation was measured using the FL 3000 fluorimeter following 2 min dark adaptation in the presence of 10 µM DCMU. The t_½_ time of Q_A_^−^ oxidations was 0.2–0.4 ms and 1.3–2.0 s for the fast and slow phases, respectively. Flash intensity was 2300 µmol photons m^−2^ s^−1^; flash duration 30 µs. The first sampling point was recorded 215 µs after the flash to minimize the contribution of the flash decay, 5 points per decade and 20% voltage of the measuring beam. Furthermore, since all measurements were performed with the same instrument and setting, the contribution of instrumental artifacts, if any, would equally occur in all the measurements. For the sake of clarity, we provide full data sets for the 0 and 35 min time point in (**A**) and the Fv values and the ratio of fast to slow decay kinetics, as a function of time of exposure to the excess light in (**B**), calculated from data such as presented in (**A**). (**C**) Same conditions as above but with the cells exposed for 17 min to various light intensities (250 to 2000 µmol photons m^−2^ s^−1^). (**D**) Same conditions as in (**A**) but the QA^−^ re-oxidation was measured at 15°C. The t_½_ time of Q_A_^−^ oxidations was 0.32–0.45 ms and 3.1–7.1 s for the fast and slow phases, respectively. Note the large rise in the extent of the fast phase and that the t_1/2_ of the fast phase was little affected by lowering the temperature from 25°C (**A–C**) to 15°C (**D–E**), whereas that of the slow phase declined by about 3-fold (compare with (A).

We examined whether the proposed changes in PSII are reflected in the kinetics of Q_A_^−^ re-oxidation. These measurements, which were performed in the presence of DCMU (thereby blocking electron transfer to Q_B_), revealed fast and slow decay components (0.2–0.4 ms and 1.3–2.0 s, respectively, **Fig. 6**A). The amplitudes of the fast phase increased significantly with the duration of excess light (2000 µmol photons m^−2^ s^−1^) treatment (**Fig. 6A** and **B**) and with light intensity (**Fig.** **6C**). Consequently, the ratio of the fast/slow phases rose in an inverse relation to the loss of Fv (**Figs. 6B**). The effect of the exposure to excess light was even more pronounced when the Q_A_ re-oxidations were measured at a lower temperature (15°C, **Fig. 6D**). The t_1/2_ of the fast phase (0.32–0.45 ms, **Fig. 6A**) was only slightly affected by the reduced temperature but it increased to 3.1–7.1 s (in different experiments) for the slower phase (**Fig. 6D** and **E**), demonstrating a stronger temperature dependence of the latter. These results indicate an increase in Q_A_ excitation de-trapping by excess light treatment, most likely reflecting a rise in electron flow *via* alternative routes, such as non-radiative charge recombination pathways.

### Excess light does not alter carotenoids absorption

**Figure 7:**
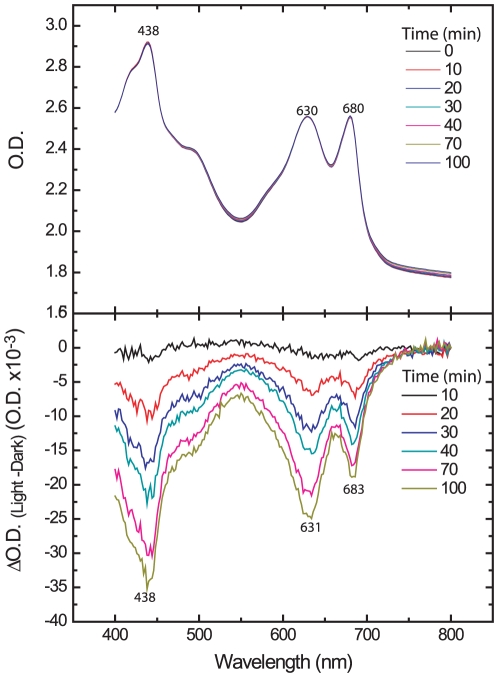
Absorption changes in high light-exposed *Microcoleus* cells. Cells suspension was exposed to 1500 µmol photons m^−2^ s^−1^ for 40 min followed by 60 min recovery at 50 µmol photons m^−2^ s^−1^, which resulted in the recovery of the Ft–Fo values (see Fig. 3B). Elapsed time (min) from the beginning of the experiment is shown in the box. **Top panel**: Absorptions in the visible range during the entire experiment. **Bottom panel**: Absorptions changes calculated from the differences between the light exposed and zero time control. O.D._800_ was used for normalization. Wavelengths are indicated where maximal absorption differences were observed. Differential absorption measurements were made using a Cary 300bio UV-visible spectrophotometer (Varian, Palo Alto, USA). Integration time was set to 0.5 s, the slit to 2 nm and the wavelength increment to 2 nm.

Lack of photoinhibition in purple bacteria, where the photosynthetic reaction center is quite similar to PSII in cyanobacteria and higher plants (with respect to the structural protein scaffold harboring the redox components), has been attributed to quenching of singlet oxygen by carotenoids [30]. In their natural habitat, dehydration after deposition of early morning dew occurs when the *Microcoleus* filaments are already exposed to excess light intensity [1]. The pioneering studies by Heber and colleagues [16] showed marked light-driven absorption changes in a desiccating cyanolichen, interpreted as representing reversible conformational alterations in PSII thus enabling fast carotenoids-mediated energy dissipation. Furthermore, tocopherol plays an important role in preventing oxidative damage and PSII depletion of carotene by singlet oxygen [31]. However, this is not the case in *Microcoleus* since only a marginal loss of absorbance at 430 nm, 631 nm and 683 nm were observed in cells exposed to excess-light for 100 min (**Fig. 7**). In addition, unlike the photosynthetic parameters, the loss of absorption did not recover even after prolonged exposure to low light. Thus it is unlikely that change in carotenoids serve as the main route of light energy dissipation in excess light-treated *Microcoleus*.

## Discussion

A rising light intensity concomitant with decreasing photochemical activity during dehydration would be expected to cause severe photodamage to the photosynthetic machinery. However, this is not the case in *Microcoleus* since rewetting of recently dried natural or artificial crusts resulted in rapid recovery of the photosynthetic activity [14],[15]. Field measurements showed a large decline in the fluorescence yield from the crusts already at light intensities approximately 1/10 that of full sunlight, even when the desert crusts were fully hydrated (**Fig. 1**). These data clearly indicate that under natural conditions the photosynthetic system responds to the rising light intensity that precedes the dehydration. Although it is possible that light-induced changes in the photosynthetic machinery is involved in the cell's preparation to the forthcoming desiccation, the resistance of *Microcoleus* photosynthesis to dehydration is distinguishable from that operating in excess light. For activation, the latter requires light intensities that saturate CO_2_-dependent O_2_ evolution, and its reversal occurs only under low light intensity (**Fig. 3B**). In contrast, reversible loss of fluorescence emission during dehydration is light-independent in *Microcoleus* [15].

We focused on the mechanisms whereby photosynthesis in *Microcoleus* is far less sensitive to excess light than model organisms [14]. Due to the very thin *Microcoleus* layer in the crusts as compared with the light path in laboratory experiments, it is hard to compare the responses to specific light intensities in the two cases. Nevertheless, in both field and laboratory experiments, including those performed with artificial crusts, the fluorescence declined in response to excess light whereas O_2_ evolution was hardly affected (**Fig. 3A**). Studies on model photosynthetic organisms revealed an intimate connection between variable fluorescence and O_2_ evolution [32], to the extent that it became a common practice to assess the rate of electron flow from the fluorescence parameters. The experiments presented here (**Fig. 3**) clearly indicated that the fluorescence decline did not reflect loss of the forward electron transport since O_2_ evolution was not affected. Studies on high-light exposed *Synechocystis* [33] and the diatom *Phaeodactylum tricornutum* [34] showed a slower decline in O_2_ evolution than of fluorescence. In both cases, however, the extent of decoupling between fluorescence and O_2_ was much smaller than in *Microcoleus* and the mechanism involved in the PSII core was not elucidated. Uncoupling fluorescence from O_2_ evolution also suggested that the primary productivity in the crusts cannot be assessed from remote sensing using fluorescence emission.

Unfortunately, it is not possible to reveal the mechanisms whereby *Microcoleus* PSII is relatively resistant to photodamage using natural crusts, therefore we focused mostly on laboratory grown artificial crusts and liquid cultures. It is unlikely that loss of fluorescence and TL was due to general damage to the thylakoid membranes since PSI activity, oxygen evolution and uptake (respiration) were not affected even when the cells were exposed to light intensities 3-fold higher than required to saturate CO_2_ fixation (**Fig. 3A**). Loss of radiative emissions (80% decline in Ft–Fo and in Fv, Figs. **3B** and **4A**) was much larger than the change in energy transfer from the phycobilisomes to PSII (16%) and also occurred under blue light (which preferentially excite chlorophyll) and in the presence of DCMU, DBMIB and FCCP (**Fig. 4C** and **D**). These observations suggested that changes such as those leading to NPQ are not the main drivers of the decline in radiative emissions, implicating changes in the core of PSII following excess light treatment. The stable isotope measurements ruled out cyclic electron transport in *Microcoleus* PSII with O_2_ serving as an electron acceptor (but see [35] for eukaryotic organisms).

Uncoupling between fluorescence yield and forward electron transport by excess light-treated *Microcoleus* could be mediated by light driven structural alteration in the core PSII [36],[37] leading to a change in the ratio between radiative and non-radiative processes in PSII [20] or core antenna quenching. Mostly due to technical reasons (very low activity of thylakoids isolated from *Microcoleus*), we are unable to conclusively distinguish between these possibilities but two sets of data support the non-radiative route. TL experiments indicated a relatively small difference (as compared with model organisms) between the redox potentials of Q_A_ and Q_B_ in *Microcoleus* (**Fig. 5**) and, as a result, a larger redox difference between Pheo and Q_A_ that would favor a non-radiative P_680_^+^ Q_A_^−^ recombination route. Neither the temperatures of maximal B and Q band emissions nor the initial oscillation pattern (**Fig. 5C**) were affected by the excess light, but nevertheless the signal amplitude declined significantly (**Figs. 3A** and **5C**). PSII centres that have already been altered by exposure to high light do not emit fluorescence or TL signals and thus changes in the energy gap between Q_A_^−^ and the electron acceptor involved in this recombination pathway cannot be recorded by non-invasive techniques.

The possibility that a non-radiative electron route is induced by excess light is also supported by the increased magnitude of the fast Q_A_^−^ re-oxidation phase with light intensity, temperature and duration of exposure (**Fig.** **6**). Vass and co-workers were able to modulate the ratio of radiative to non-radiative recombination in PSII by mutating residues that participate in the binding of Pheo [5],[20]. They concluded that the probability for radiative/non-radiative recombination is under control of the redox potentials of the Pheo and Q_A_. It is important to note that the change in redox the mutants produced is not reversible while in *Microcoleus* it can readily reverse under low light conditions.

Taken together, the data presented here are all consistent with a model whereby activation by excess light of a non-radiative recombination route, bypassing the regeneration of the primary charge separated pair (P_680_^+^/Pheo^−^), allows for the reduction of P_680_^+^ without the formation of damaging active oxygen species [5],[7],[20],[26]. At this time we do not know the exact electron route from Q_A_ to P^+^ that competes with the formation of ^3^P_680_ by charge recombination, thereby reducing the generation of ^1^O_2_ and oxidative damage to PSII. Loss of radiative charge recombination even in the presence of DCMU, DBMIB and FCCP implies that an increase in the back electron flow, when the forward one is inhibited, may affect the ratio of different charge recombination pathways [5],[20]. The route of non-radiative back electron flow could involve the third quinone binding Q_c_ site [38],[39] and cytochrome *b*_559_ [40]. The mechanism(s) whereby this route is activated only under excess light, thereby minimizing futile electron flow at limiting light intensity, remains to be elucidated; light activated structural change in a photosynthetic reaction center was recently reported [36],[37].

Earlier suggestions of PSII cyclic electron flow routes involved the reduction of O_2_ [22],[23],[41] or cycling within PSII [18],[42],[43],[44]. Clearly the proposed non-radiative pathway differs substantially from those that involve reduction of plastoquinone or oxygen since it is insensitive to DCMU and DBMIB (**Figs. 4** **and** **6**) and accelerated reduction of oxygen was not observed.

An alternative PSII photo-protective mechanism was proposed based on the observation that close reaction centers (reduced Q_A_) may cause slowing down of primary charge separation [45]. However, the contribution of such a mechanism is likely to be small in the case of *Microcoleus* since loss of variable fluorescence in excess-light treated cells was detected during either continuous light or after prolonged dark adaptation (**Fig. 3B**) where Q_A_^−^ was already oxidized.

A protective electron transfer route as reported here may also function under specific conditions in certain cyanobacterial mutants, albeit to a lesser extent than in *Microcoleus* [33]. The *Microcoleus* capability presented here is the first report showing light-induced changes in PSII core in a naturally growing organism. Considering the ancestor cyanobacterial evolutionary origin of the chloroplast, it is not clear why an effective mechanism that helps *Microcoleus* to minimize photoxidative damage does not seem to operate in higher plants.

## Materials and Methods

### Field measurements

Sand crusts were taken from the Nizzana field site in Petri dishes 1–3 days before measurements. The plates were placed outdoors on a special stand that enabled us to accurately and repeatedly direct the light guide of the PAM to exactly the same spot. Fluorescence emitted from 4 plates was measured using a PAM 2500 (Walz, Effertlich, Germany). Measurements were carried out every 10 min between midnight and 09:00. Two plates were hydrated by spraying water to prevent dehydration.

### Cell cultivation

*Microcoleus* filaments [15] were cultivated in BG11 medium at 25°C under fluorescent light (20–30 µmol photons m^−2^ s^−^). The aggregated cells suspension were homogenized in BG11 medium and incubated with gentle rotatory shaking (200 ml in 500 ml Erlenmeyer flasks) for at least 2–3 days before the experiments. The extensive EPS layers surrounding the *Microcoleus* filaments [15] hindered our attempts to isolate thylakoids exhibiting significant photosynthetic activity using conventional methods. Hence all data presented here were obtained using cell suspensions and non-invasive techniques. Protein synthesis inhibitors such as chloramphenicol could not be used to estimate the light-induced loss of proteins since their penetration was not reproducible, possibly due to the variable amounts of EPS. The small variability in the results obtained in various experiments is attributed to the differences in the rate of cell division within the filaments aggregates, which resulted in a mixture of dividing and stationary phase cell populations. Due to the entangled filaments that could not be dispersed completely by homogenization, it is difficult to dilute the cells suspension exactly to a given concentration.

### Artificial crusts of *Microcoleus*

*Microcoleus* suspensions (5 ml, 0.25 O.D_(678 nm)_ were filtered through nitrocellulose filters (0.4 µm) by mild vacuum suction to form an equally distributed artificial film. Crusts were formed following incubation (25°C, 30 µmole photons m^−2^ s^−1^) in Petri dishes on filter paper maintained wet by periodic additions of water, and continued to grow in thickness for up 48 h (about 0.1–0.2 mm thickness in the wet state). The crusts could be removed from the filter as a very thin film by gentle scraping, re-suspended in BG11 medium and dispersed again to a homogeneous filaments suspension by passing through a narrow syringe tip.

### O_2_ exchange

Clark type O_2_ electrode (PS2108, Passport dissolved O_2_ sensor Roseville, CA, USA) was used. Cells suspended in fresh BG11 medium containing 2 mM NaHCO_3_ were incubated in a temperature-controlled Perspex holder (optical path 1.5 cm, 25°C). The extent of light dependent ^18^O_2_ uptake relative to water splitting in PSII (indicated by ^16^O_2_ production) was measured using a membrane inlet mass spectrometer (QMS-200, Pfeiffer vacuum, Germany) [25]. Cells suspended in BG11 media were exposed at 25°C to either 30 or 1500 µmol photons m^−2^ s^−1^. Gross O_2_ evolution was assessed from ^16^O_2_ formation and uptake of ^18^O_2_ was used to measure oxygen consumption. The rate of ^18^O_2_ reduction in the presence of DCMU was identical to that observed in darkness. These data do not support a significant cyclic electron transfer in PSII of *Microcoleus* with O_2_ serving as the electron acceptor.

### Thermoluminescence emission

**Figure 8:**
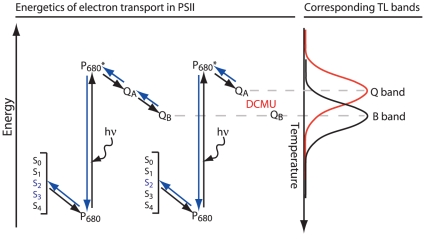
A schematic presentation of electron transport within PSII and the resulting TL bands. The Q_A_ and Q_B_ quinones, P_680_ and the Mn cluster S-states are shown. Forward electron transport is represented by plain arrows and back electron transport by blue arrows. Changes in the peak temperature observed in TL experiments can be used as an estimate for the recombination energy of the Q_B_^−^S_2_/Q_B_S_1_ and Q_B_^−^S_3_/Q_B_S_2_ (B-band) or Q_A_^−^S_2_/Q_A_S_1_ (Q-band) redox pairs. To observe the Q band emission, reduction of the Q_B_ site was blocked by herbicides that bind specifically to this site. The electron released from P_680_ by light excitation at subzero temperatures can reach the Q_A_ site and, in the presence of herbicides that bind to the Q_B_ site, recombine upon warming with the oxidized S_2_ state by back electron flow producing the Q band. Since the energy gap between Q_A_^−^ and P_680_ *^+^* is smaller than that from Q_B_^−^ to P_680_ *^+^* (B band), lower activation energy is required for this recombination and thus the Q band is observed at lower temperatures.

Thermoluminescence (see the scheme in **Fig. 8**) was recorded as described [27]. Samples (0.4 ml, 4–5 µg chlorophyll) were dark-adapted at 25°C for 2 min, rapidly frozen to −22°C and excited by saturating light flashes (3 µs, xenon arc discharge). The samples were then heated at a rate of 0.6°C s^−1^ to 50°C while counting photon emissions (B band). For detection of the Q band, the herbicides DCMU or ioxynil (Sigma, Aldrich, Germany), which bind to the Q_B_ site, were added before dark-adaptation at concentrations completely inhibiting oxygen evolution (5 and 35 µM, respectively).

### Spectroscopy

Since EPS encapsulating the entangled filaments hindered extraction of the pigments, chlorophyll content was estimated from absorption spectra. The maximal absorption of phycobilisomes (at 625 nm) was generally equal (+/− 5%) to that of chlorophyll *a* (at 678 nm). The cells suspensions absorption was in the range of 0.8 O.D. at 678 nm.

### Fluorescence rise/decay kinetics and P700 photo-oxidation

These were measured with a specially designed pump-probe measuring head for the FL3000 fluorimeter providing blue and orange light excitation (PSI, Brno, Czech Republic), and an IMAG-MAX PAM providing blue light excitation (Walz, Effertlich, Germany). A JTS10 kinetic spectrophotometer (Bio-Logic, Calix, France) was used for P_700_ measurements (530 nm, 590 µmole photons m^−2^ s^−1^, 5 sec illumination and detection at 705 nm).

Measurements of the effects of FCCP and DBMIB on light-induced loss of variable fluorescence were performed using the FL3000 fluorimeter. Cells corresponding to 0.9–1.0 OD_678 nm_, 5 mm optical path, light excitation intensity was 1800 µmol photons m^−2^ s^−1^ for 30 s. Pre-illumination of cells suspension to induce loss of fluorescence emission was carried out using the same cell concentration, 2.5 cm optical path, and 2000 µmol photons m^−2^ s^−1^ for various durations as indicated in the figure legends. FCCP (10 µM) or DBMIB (0.15 µM) were added at the onset of illumination. The low DBMIB concentration was selected since it is known to quench fluorescence. At the concentration used here it reduced the variable fluorescence by 15%, thus allowing detection of the fluorescence loss resulting from exposure to the excess light. Cell respiration was not affected during the exposure to high light in presence of these inhibitors but oxygen evolution was severely inhibited >75%.

### Excess light treatments

High illumination treatments were carried out with cell suspension in the O_2_ electrode chamber deliberately kept open during the interim periods of light treatments in order to minimize the rise in O_2_ concentration. When larger amounts of cells were required, light exposure was performed in a temperature-controlled glass container 2.2 cm optical path, with 50 ml of cells. The oxygen level was lowered to about 30–40% of air saturation by bubbling with nitrogen. The chamber was then closed, the cells dark-adapted for 2 min while measuring respiration and samples were then taken for TL and fluorescence emission measurements. This was followed by measurements of O_2_ evolution under the selected light intensity. Longer dark adaptations (up to 5 min) did not change the fluorescence emission parameters or O_2_ evolution of excess light-treated samples. Interruptions of illumination for less than 5 min did not induce detectable changes in oxygen evolution.
